# p53 Suppresses Tetraploid Development in Mice

**DOI:** 10.1038/srep08907

**Published:** 2015-03-10

**Authors:** Takuro Horii, Masamichi Yamamoto, Sumiyo Morita, Mika Kimura, Yasumitsu Nagao, Izuho Hatada

**Affiliations:** 1 Laboratory of Genome Science, Biosignal Genome Resource Center, Institute for Molecular and Cellular Regulation, Gunma University, 3-39-15 Showa-machi, Maebashi, Gunma 371-8512, Japan; 2 Advanced Scientific Research Leaders Development Unit, Gunma University, 3-39-22 Showa-machi, Maebashi, Gunma 371-8511, Japan; 3 Medical Research Center, Jichi Medical University, 3311-1 Yakushiji, Shimotsuke, Tochigi 329-0498, Japan

## Abstract

Mammalian tetraploid embryos die in early development because of defects in the epiblast. Experiments with diploid/tetraploid chimeric mice, obtained via the aggregation of embryonic stem cells, clarified that while tetraploid cells are excluded from epiblast derivatives, diploid embryos with tetraploid extraembryonic tissues can develop to term. Today, this method, known as tetraploid complementation, is usually used for rescuing extraembryonic defects or for obtaining completely embryonic stem (ES) cell-derived pups. However, it is still unknown why defects occur in the epiblast during mammalian development. Here, we demonstrated that downregulation of p53, a tumour suppressor protein, rescued tetraploid development in the mammalian epiblast. Tetraploidy in differentiating epiblast cells triggered p53-dependent cell-cycle arrest and apoptosis, suggesting the activation of a tetraploidy checkpoint during early development. Finally, we found that p53 downregulation rescued tetraploid embryos later in gestation.

One important mechanism for functional innovation during evolution is the duplication of genes and even entire genomes. Accumulating evidence shows that during vertebrate evolution entire genomes underwent two rounds of duplication through tetraploidy^1^. Whole genome duplication quickly produced enormous numbers of new genes in early vertebrates (such as the tumour suppressor gene, *p53*) and promoted rapid evolution^2^,^3^. Furthermore, it is widely recognised that 30–80% of plants are polyploid, having undergone polyploidisation events, including tetraploidy, throughout their evolutionary history^4^. Interestingly, artificial tetraploidisation between the same or different species is a very popular technique used in plants for breeding improvement. In lower vertebrates, such as fish and amphibians, natural tetraploidy and the induction of artificial tetraploidy are also common phenomena^5^,^6^. By contrast, tetraploidy has not been usually observed in higher vertebrates such as reptiles, birds, and mammals. In mammals, tetraploidy is generally incompatible with normal development, and although spontaneous duplication of the genome does occur in approximately 0.1–7.1% of fertilizations^7^, mouse tetraploid embryos die in early development^8^,^9^. Snow reported full-term development of mouse tetraploid embryos in the 1970s^10^,^11^, but this experiment was never replicated by other researchers. Human tetraploid embryos generally abort spontaneously and are usually characterised by empty chorionic sacs, lacking embryonic tissue^12^.

Experiments with diploid/tetraploid aggregation chimeric mice clarified that while tetraploid cells are excluded from the epiblast derivatives of mid-gestation embryos^8^,^13^,^14^, diploid embryos with tetraploid extraembryonic tissues can develop to term. Today, this method, known as tetraploid complementation, is usually used for rescuing extraembryonic defects^15^ or for obtaining completely embryonic stem (ES) cell-derived pups. Thus, any developmental defects in the tetraploid embryos will not be due to trophectoderm defects but to defects of the epiblast, which is derived from the inner cell mass (ICM). However, there has not been reported why crucial defects of the epiblast occur during mammalian development. Here, we demonstrate that p53, a tumour suppressor protein, suppresses tetraploid development in mice.

## Results

### Most mouse tetraploid embryos die at the egg cylinder stage

**Figure 1:**
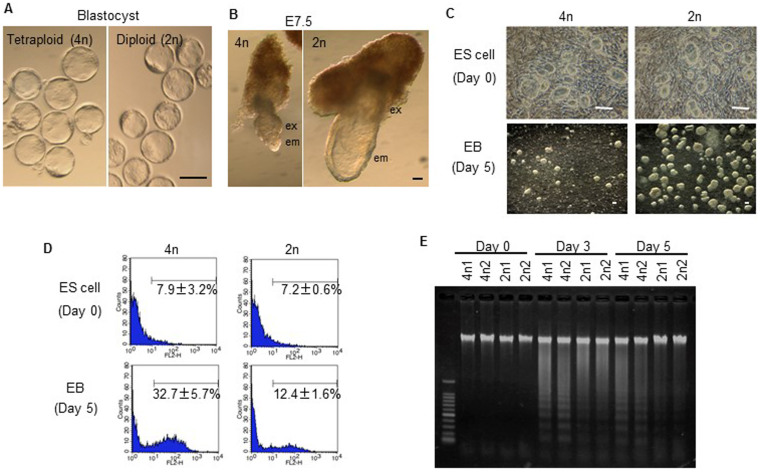
Tetraploid ES cells show apoptosis after differentiation induction. (A) Tetraploid (4n) and diploid (2n) embryos are morphologically similar at the blastocyst stage. (B) Epiblast-derived embryonic tissue is retarded in tetraploid embryos at E7.5. (C) Undifferentiated tetraploid and diploid ES cells appear morphologically similar (top). Following differentiation induction, tetraploid ES cells form poorly developed EBs compared to diploid ES cells (bottom). (D) FACS analysis for active caspases before and after differentiation induction. The numbers show the percentages of caspase-positive cells. (E) Detection of apoptosis using the DNA ladder method for tetraploid ES cells lines, 4n1 and 4n2 and diploid ES cell lines, 2n1 and 2n2.

In the current study, tetraploid embryos were produced by electrofusion of blastomeres from two-cell embryos. The tetraploid embryos developed normally to the blastocyst stage; however, both embryonic and extra-embryonic tissues were reduced, and exhibited morphological abnormalities especially in embryonic tissues at embryonic day (E) 7.5 (Fig. 1A,B). After this stage, embryo size was reduced, the development of epiblast-derived tissue was delayed significantly, and living embryos were rarely obtained beyond E9.5. Early abnormalities in tetraploid embryos suggest that tetraploidy might reduce the pluripotency or proliferation of ICM-derived cells. To determine the events that occurred during the development of tetraploid ICM-derived embryonic cells, tetraploid ES cell lines derived from the ICM of tetraploid blastocysts were established as the differentiation of ES cells into embryoid bodies (EBs) *in vitro* mimics events that occur during postimplantation.

### Tetraploid ES cells die by apoptosis after induction of differentiation

Tetraploid ES cell lines (B6-EGFP × PWK) were established with the low efficiency compared with normal diploid ES cells (tetraploid 22% vs diploid 80%). Our tetraploid ES cell lines maintained tetraploidy (passage 5) (Fig. S1A–D), and although they were larger than the diploid ES cells due to an increase in the total amount of DNA, the proliferation speed between the two cell types did not vary (Fig. S1E,F), and therefore did not cause the developmental defects seen in tetraploid embryos. Tetraploid ES cells expressed the same pluripotency markers as diploid ES cells (Fig. S2), but at slightly higher (*Oct4* and *Sox2*) or lower (*Stella* and *Fgf5*) levels than in diploid ES cells. Nevertheless, tetraploid ES cells retained the representative morphology of undifferentiated diploid ES cells, indicating that this gene expression pattern in tetraploid ES cells is compatible with the undifferentiated state. Thus, there seemed to be no critical differences between undifferentiated tetraploid and diploid ES cells. By contrast, inducing ES cell differentiation demonstrated that tetraploid ES cells underwent dramatic levels of cell death and poor EB formation compared to diploid ES cells (Fig. 1C). Both fluorescence activated cell sorting (FACS) analysis for active caspases and apoptotic DNA ladder analysis indicated that most tetraploid ES cells died by apoptosis immediately after the induction of differentiation (Fig. 1D,E).

### p53-dependent apoptosis occurs in differentiating tetraploid ES cells

**Figure 2:**
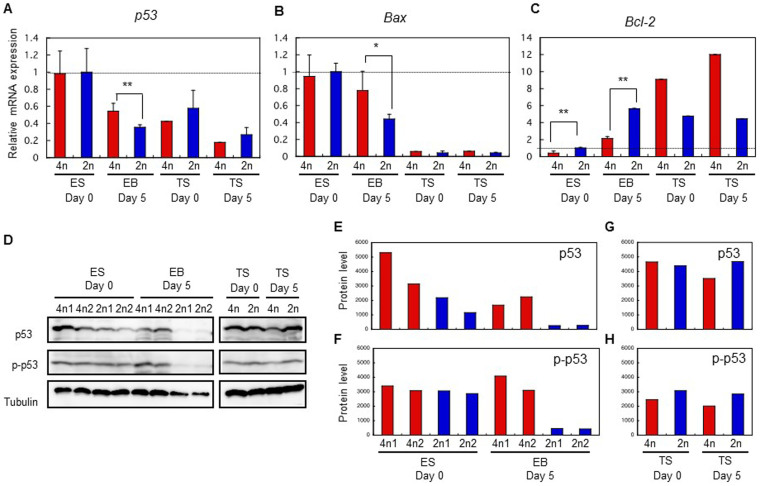
Constitutive activation of p53 in tetraploid ES cells after differentiation induction. Quantitative real-time RT-PCR for (A) *p53*, (B) *Bax*, and (C) *Bcl-2* in ES and TS cells before and after differentiation. Data were normalized to the expression of *Gapdh*. Genes showing significant differences between tetraploid and diploid cells (*p < 0.05; **p < 0.01). (D) Immunoblots analysis of p53, p-p53(Ser18), and tubulin expression in ES and TS cells. For p53 and p-p53, 1 × 10^5^ cells were loaded into each lane; for tubulin, 5 × 10^3^ cells were loaded into each lane. The gels have been run under the same experimental conditions. Full-length blots/gels are presented in Figure S3.

We hypothesised that p53-dependent apoptosis (p53/Bax/cytochrome c/caspase-dependent pathway) occurs in tetraploid ES cells after differentiation induction. p53, known as ‘the guardian of the genome’, is a multifunctional transcription factor that stimulates both cell-cycle arrest and apoptosis by inducing genes such as *p21*^16^ and *Bax*^17^. By contrast, Bcl-2 protein forms heterodimers with Bax and inhibits apoptosis^18^. The present study showed that the levels of *p53* mRNA in tetraploid and diploid ES cells under undifferentiated conditions were similar; however, once ES cells became differentiated, the p53 expresson level in tetraploid ES cells was higher than in diploid ES cells (Fig. 2A). In addition, the expression of apoptosis promoter *Bax* increased, whereas that of the apoptosis inhibitor, *Bcl-2*, decreased (Fig. 2B,C) in differentiated tetraploid ES cells compared with diploid ES cells. By contrast, tetraploid trophoblast stem (TS) cells consistently showed low expression of *Bax* mRNA and high expression of *Bcl-2* mRNA, indicating that p53-dependent apoptosis was not triggered in TS cells (Fig. 2B,C); thus the mRNA assay indicated that p53/Bax/cytochrome c/caspase-pathway was activated in differentiating tetraploid ES cells. These findings were confirmed by immunoblot analysis of p53 and phosphorylated-p53 (p-p53) expression. Phosphorylation of p53 (Ser18 in mice and Ser15 in humans) is necessary to activate the p53/Bax/cytochrome c/caspase-pathway^19^. The results of the immunoblot analysis indicated that the total amount of p53 in tetraploid ES cells was higher than in diploid ES cells both before and after differentiation (Fig. 2D,E). However, far more p-p53 protein was present in tetraploid ES cells than in diploid ES cells after differentiation, although the p-p53 level was did not differ to undifferentiated conditions (Fig. 2D,F). There was no difference in p53 and p-p53 levels between tetraploid and diploid TS cells (Fig. 2D,G,H).

Following differentiation, the well known pluripotency markers, *Oct4* and *Nanog*, are generally downregulated by DNA methylation of their promoter and/or enhancer regions^20^,^21^; however, expression of these genes was not sufficiently downregulated in our tetraploid ES cells (Fig. S4A), while their promoter and enhancer regions remained hypomethylated (Fig. S4B,C). This result shows that small populations of tetraploid ES cells, that had not undergone differentiation, survived after differentiation induction.

### Tetraploid embryos die by p53-dependent apoptosis

**Figure 3:**
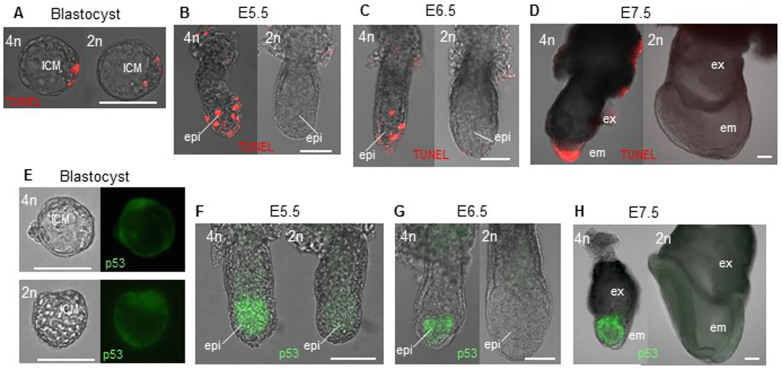
Tetraploid embryos show p53-dependent apoptosis after differentiation induction. (A–D) TUNEL staining for tetraploid and diploid embryos. (E–H) p53 expression in tetraploid and diploid embryos. epi, epiblast; em, embryonic tissue; ex, extra-embryonic tissue. Scale bars: 0.1 mm.

We expected that murine tetraploid development is also suppressed by p53-dependent cell-cycle arrest and cell death in the egg cylinder epiblast. Therefore, we used the TUNEL assay to test whether apoptosis could account for the tetraploid embryo phenotype. At the blastocyst stage, a few ICM cells in both tetraploid and diploid embryos were TUNEL-positive (Fig. 3A). At this stage, ICM cell death by apoptosis is a common phenomenon for eliminating abnormal cells^22^; however, there was no significant difference between tetraploid (3.25 +/− 0.96, *n* = 15) and diploid (2.75 +/− 0.50, *n* = 7) blastocysts. At E5.5–6.5, apoptosis was observed in the visceral endoderm and epiblast in all tetraploid embryos (Fig. 3B,C) but there was very little apoptosis in diploid embryos. Tetraploid embryos at E7.5 showed apparently retarded epiblast-derived tissues with TUNEL-positive cells, which were not observed in diploid embryos (Fig. 3D). In accordance with TUNEL staining, p53 expression was elevated in tetraploid epiblast cells at E5.5–7.5 (Fig. 3E–H) but not in blastocysts and trophoblast cells. Both ICM and trophectoderm seem to be p53-positive in tetraploid and diploid blastocysts, and both tetraploid and diploid trophoblast cells showed low expression of p53. Thus, excessive p53 expression in tetraploid embryos could cause cell-cycle arrest and apoptosis around E5.5–7.5, resulting in poor embryonic tissue formation.

### p53 downregulation improves tetraploid development

**Figure 4:**
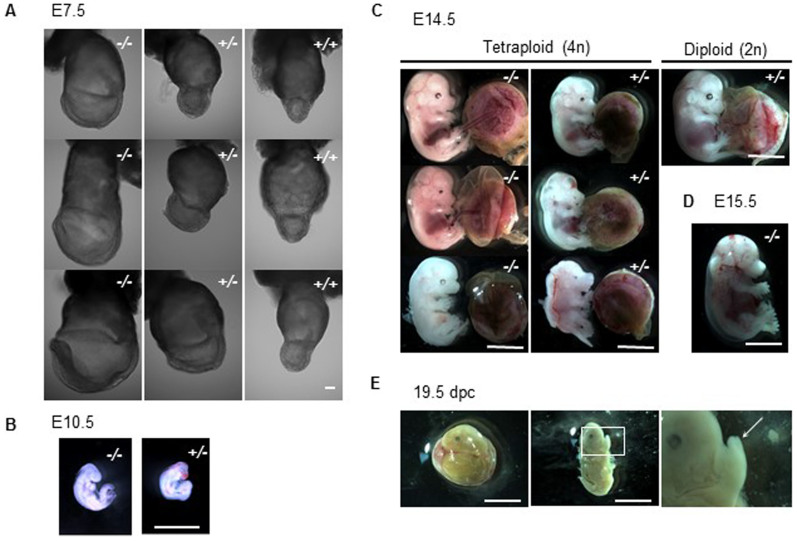
p53 downregulation improves developmental potential of tetraploid embryos. (A) Tetraploid embryos at E7.5. *p53*^+/+^ embryos (right) show retarded embryonic tissues while most *p53^−/−^* and portions of *p53^+/−^* embryos appear normal. (B) Only *p53*^+/−^ and *p53^−/−^* embryos were recovered at E10.5. (C) Tetraploid (4n) and diploid (2n) embryos at E14.5. Tetraploid embryos often showed smaller eyes and forebrain vesicles than normal diploid embryos. (D) A *p53*^−/−^ tetraploid embryo at E15.5, lacking heart-beat. (E) Tetraploid embryos were recovered at 19.5 days of pregnancy, but development had stopped around E14.5, based on finger morphology (arrow). Scale bars: 0.1 mm (A), 1 mm (B), 5 mm (C–E).

Next, we examined whether p53 downregulation in tetraploid embryos could overcome the apoptosis observed in the epiblast. The developmental potential of tetraploid embryos, derived from *p53*^+/−^ × *p53*^+/−^ fertilised embryos transferred to pseudopregnant recipients was examined (Table 1). At E5.5–7.5, embryos with all three *p53* genotypes were recovered; however, epiblast-derived-tissues of all *p53*^−/−^ and most of the *p53*^+/−^ tetraploid embryos appeared well grown compared to the *p53^+/+^* embryos at E7.5 (Fig. 4A and Fig. S5), indicating that p53 is a key regulator of tetraploid development. The morphological difference among the same genotype was observed, indicating phenotypic variation at E7.5. In addition, TUNEL positive cells were reduced in these p53-downregulated embryos (Fig. S5). Interestingly, *p53^+/−^* embryos at E5.5 showed two phenotypes on the whole; one is TUNEL-positive (4/8, 50%), and the other is TUNEL-negative (4/8, 50%). This phenotypic difference in *p53^+/−^* embryos could cause the higher survival ratio of *p53^−/−^* embryos than that of *p53^+/−^* embryos at E10.5–14.5 (Table 1), comparing to the theoretical frequency of appearance (*p53^+/−^*:*p53^−/−^* = 1:2).

**Figure 5:**
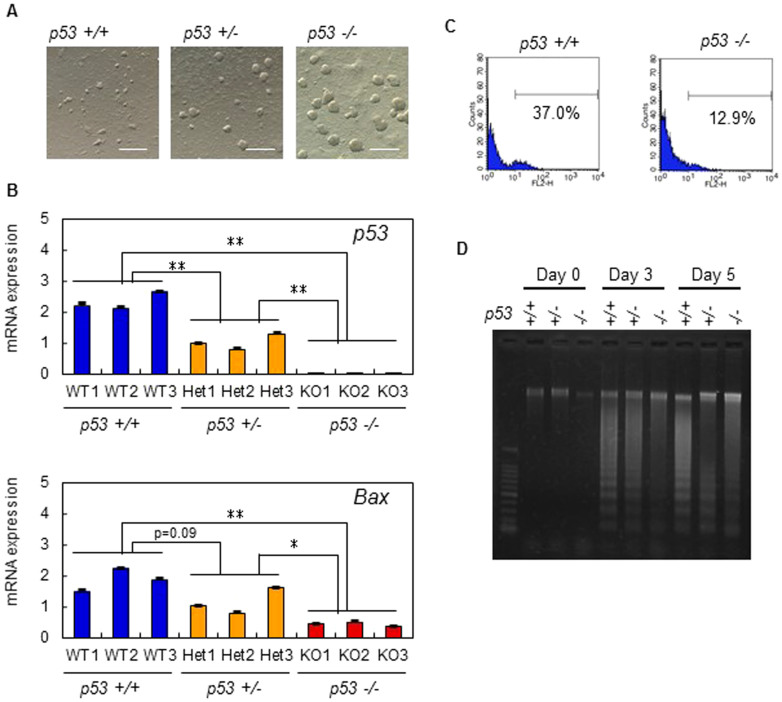
Downregulation of *p53* improves EB formation of tetraploid ES cells by escaping apoptosis. (A) EB formation (day 5) of *p53+/+*, *p53+/−* and *p53−/−* tetraploid ES cell lines. *p53*-deficient tetraploid ES cells formed well-developed EBs. (B) Relative mRNA expression of *p53* and *Bax* of EBs (day 5) derived from *p53+/+*, *p53+/−* and *p53−/−* tetraploid ES cell lines. Genes showing significant differences between tetraploid and diploid ES cells (*p < 0.05; **p < 0.01). (C) FACS analysis for active caspases before and after differentiation induction. Percentages show the ratio of caspase-positive cells. (D) Detection of apoptosis using the DNA ladder method. Scale bars: 0.5 mm.

Embryos were harvested next at E10.5, because tetraploid embryos are not normally observed beyond E9.5^8^,^9^. Surprisingly, more than 20% of apparently normal tetraploid embryos implanted were recovered at this stage, and all of them were p53-downregulated embryos (Fig. 4B and Table 1). At E14.5, more than 20% of tetraploid embryos implanted were recovered (Fig. 4C and Table 1). These embryos maintained tetraploidy almost completely, indicating that tetraploid embryos did not become diploid or aneuploid following the downregulation of p53 (Fig. S6). In rare cases, for example, in mice with chromosome translocations on a specific genetic background, tetraploid embryos were reported to develop beyond E9.5 at a very low frequency; however, none reached E14.5 and all possessed characteristic craniofacial abnormalities^23^. Our tetraploid embryos at E14.5 also had craniofacial abnormalities, such as smaller eyes (~90%) and abnormal forebrain vesicles (~20%) compared to normal embryos, which agreed with previous reports^23^. In the current study, one *p53*^−/−^ embryo was recovered at E15.5, (Fig. 4D). On the whole, this embryo showed normal morphology, but its heart had stopped beating. At 19.5 days of pregnancy, no live embryos were recovered; however, the presence of retarded embryos suggested that pregnancy had probably progressed to around E14.5 (Fig. 4E). In this experiment, *p53*^+/−^ embryos showing a 50% reduction in p53 alleles survived to a much later stage of gestation than *p53^+/+^* embryos. In an *in vitro* differentiation experiment, we demonstrated that p53 downregulation in tetraploid ES cells prevented apoptosis during differentiation and improved formation of EBs (Fig. 5A). Expression of a *p53* and its target *Bax* was downregulated in *p53^+/−^* and *p53^−/−^* ES cells after differentiation (Fig. 5B). Moreover, p53 deficient EBs showed downregulation of apoptosis markers (Fig. 5C,D). Thus, taken together, the data show that p53 depletion greatly improves tetraploid embryo development.

### Tetraploid embryos rescued by diploid ES cells develop to term

**Figure 6:**
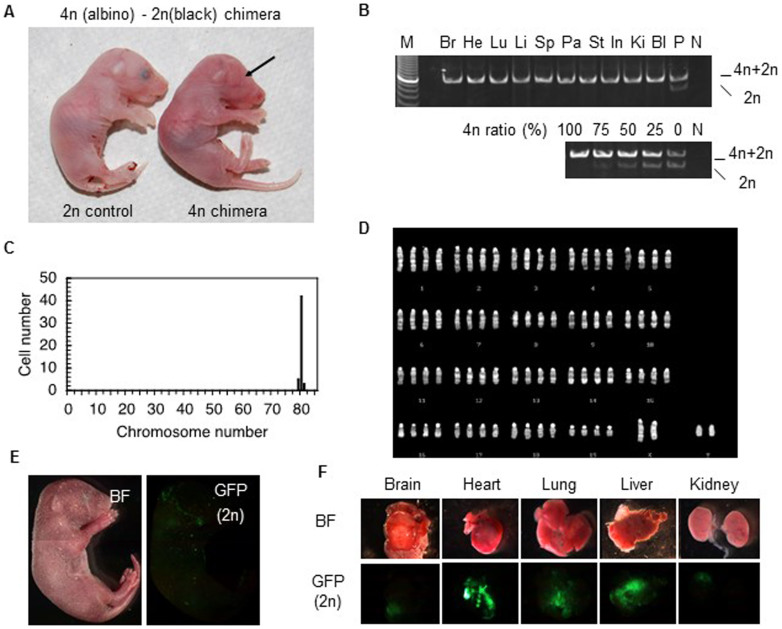
Almost completely tetraploid mice were born via diploid ES cell rescue. (A) Newborn tetraploid chimera generated by injecting diploid male ES cells (WBB6F1-*W/W^v^*) into a p53-deficient tetraploid blastocyst (right). Diploid (WBB6F1-*W/W^v^*) newborn shown as a control (left). Lack of eye pigmentation in chimera (arrow) indicates dominant contribution of tetraploid cells. (B) PCR for tetraploid and diploid contributions confirms tetraploidy. Br, Brain; He, Heart; Lu, Lung; Li, Liver; Sp, Spleen; Pa, Pancreas; St, Stomach; In, Intestine; Ki, Kidney; Bl, Bladder; P, positive control (*W/W^v^* ES cells); N, negative control (water). (C) Chromosome analysis suggests that this chimera is almost completely tetraploid. (D) Karyotype analysis of chimeric embryo. (E) Tetraploid chimera generated by injecting diploid ES cells (GFP positive) into a p53-deficient tetraploid blastocyst (GFP negative). (F) Tetraploid-derived cells (GFP negative) contributed highly to various organs (~74.4%). BF, bright field.

In tetraploid-diploid aggregation chimeras, tetraploid cells are generally restricted to the extraembryonic tissues^8^,^9^; however, Eakin *et al*. reported that tetraploid cells contributed sporadically to chimeras, contributing <1% of the total cell number in the embryo at E10.5^9^. To examine whether p53 downregulation increases the chimeric contribution of tetraploid cells, we generated chimeras by injecting wild-type diploid ES cells into *p53*-deficient tetraploid blastocysts. Thirteen pups were harvested by caesarean section at 19.5 days of pregnancy, six of which contained tetraploid embryo-derived cells (Table 2). Surprisingly, four of the chimeras had a high contribution of tetraploid cells. One, a live male tetraploid chimera (ID: 110416CS2) injected with male diploid ES cells (WBB6F1-*W/W^v^*; black eyes) was almost completely tetraploid (Fig. 6A). PCR analysis did not detect any contribution from diploid cells in ten tissues examined (>99.9%, Fig. 6B). Additionally, chromosome analysis of epithelial cells of back skin origin also showed no diploid cell contribution (Fig. 6C,D). This live chimera appeared anatomically normal (Fig. 6A), and had a normal birth weight (1.24 g), similar to control diploid newborns (1.04–1.31 g); however, it died shortly after birth. Another chimera (ID: 111109CS1) showed 22 ~ 68% tissue contribution of tetraploid cells (brain 68%, heart 36%, lung 53%, liver 47%, spleen 31%, pancreas 22%, stomach 32%, intestine 42%, bladder 22%, and testis 42%). Fluorescent microscopy analysis revealed that tetraploid chimera (ID: 110522CS4) injected with female diploid ES cells (GFP positive) showed 4 ~ 74% (brain 4%, heart 57%, lung 74%, liver 32%, spleen 37%, pancreas 51%, stomach 40%, intestine 57%, kidney 40%, bladder 24%, and testis 52%) tissue contribution from tetraploid cells (Fig. 6E,F). This chimera had a male external genital organ and testes whereas the introduced diploid ES cells were female, indicating that dominant tetraploid cells determined the sexual fate of this chimera. Chimeras with low contribution of tetraploid cells have now survived for two years. Survived chimeras were fertile, but all offsprings were derived from diploid origins.

## Discussion

The results of this study demonstrated that p53 suppressed tetraploid development in mice. Undifferentiated embryos and ES cells do not have a tetraploidy checkpoint; however, tetraploidy in differentiating cells triggers p53-dependent apoptosis, suggesting that a tetraploidy checkpoint is activated during differentiation. *p53*-deficient tetraploid embryos developed to E14.5–15.5, whereas wild-type tetraploid embryos stopped developing around E8.5. Moreover, when diploid cells contributed to prenatal development, we discovered that, in some cases, tetraploid embryo-derived live mice were born. This result was extremely surprising because, traditionally, tetraploid-diploid aggregation chimeras are used to obtain only diploid embryo-derived mice^13^,^14^. The present study is the first to demonstrate that, using diploid cell rescue, murine tetraploid cells are capable of full-term development.

Several reports suggest that in somatic cells, cell-cycle progression in G1 phase following a failure in cell division is blocked by a p53-dependent tetraploidy checkpoint^24^,^25^. Margolis et al. proposed that the tetraploidy checkpoint prevents the proliferation of tetraploid cells in somatic tissues^26^. By contrast, recent reports show that the G1 arrest and apoptosis of tetraploid cells is caused by high concentrations of drugs used to abort cytokinesis. Indeed, when low concentrations of actin-depolymerizing drugs were used, tetraploid cells survived and progressed to the next phase of the cell cycle^27^,^28^; however, the survival rate of tetraploid cells is generally low, and most cells arrest in G1. In this regard, Storchova and Kuffer suggested two possibilities as to why tetraploid cells die through p53-dependent G1 arrest and apoptosis^29^. One possibility is that abnormal mitosis seriously damages the mitotic apparatus and/or cytoskeleton, which, in turn, activates the checkpoint response. The second possibility is that aberrant mitosis can cause DNA damage, which then triggers G1 arrest and cell death. Either way, p53 is one of the key factors involved in tetraploid survival in somatic cells.

However, the present study suggests that the tetraploidy checkpoint also plays a role in mammalian development. Abnormal mitosis in tetraploid embryos and ES cells could damages mitotic apparatus, cytoskeleton or DNA, which then triggers p53-upregulation, although this regulation still depends on ambiguous manner. For example, a *p53^+/−^* embryo harbouring a 50% reduction in *p53* alleles can overcome this checkpoint. Even in diploid embryos, p53 is present at low levels, although it does not trigger apoptosis^30^, indicating that tetraploid development is determined by subtle changes in p53 expression. In fact, the morphological difference among tetraploid embryos (Fig. 4A) indicates phenotypic variation at E7.5. In addition, most human tetraploid embryos abort spontaneously and are usually characterised by empty chorionic sacs that lack embryonic tissue^12^; however, several studies report liveborn tetraploid human infants^31^,^32^,^33^, suggesting that the stringency of the tetraploidy checkpoint differs even among mammalian species.

The present study also showed that inducing the differentiation of tetraploid ES cells triggers cell death. Indeed, much higher levels of p-p53 protein were detected in differentiated tetraploid ES cells than in differentiated diploid ES cells, which led to higher expression of the *Bax* gene, which is a p-p53 target gene. Furthermore, expression of mRNA for the apoptosis suppressor, *Bcl-2*, was markedly upregulated in diploid ES cells after differentiation, whereas the degree of upregulation in tetraploid ES cells was less marked. These data may explain why the p53-dependent tetraploidy checkpoint is activated by differentiation induction. Nevertheless, the total amount of p53 protein in tetraploid ES cells was much higher than in diploid ES cells, even when undifferentiated and thus, it is important to discuss why tetraploidy cell death does not occur in ‘undifferentiated’ tetraploid ES cells even though they express high levels of p53 protein. In this respect, it is interesting to note that p53 checkpoint pathways in undifferentiated ES cells are compromised by factors that affect the nuclear localization of p53 and by the loss of downstream factors that are necessary for the induction of cell-cycle arrest^34^. In addition, undifferentiated ES cells have such a short G1 phase that p53 is not able to trigger cell-cycle arrest^35^. Taken together, these reports suggest that undifferentiated ES cells do not have a p53-dependent checkpoint. On the other hand, downregulation and conformational change of p53 has occurred to inactivate p53 function during *in vitro* differentiation of ES cells^36^. If this process is not conducted appropriately in tetraploid ES cells, cell-cycle arrest and apoptosis could be induced during differentiation process. Therefore, once the differentiation signal is initiated, tetraploid ES cells with higher expression of p53 will be much more susceptible to apoptosis.

In addition, immunohistochemical analysis of post-implantation embryos and expression analysis of TS cells indicated that the tetraploidy checkpoint does not exist in extraembryonic lineages. For example, the immunohistochemistry results showed that the amount of p53 protein in trophoblasts in tetraploid embryos was much less than in epiblasts at E5.5–7.5. Moreover, immunoblot analysis revealed that both p53 and p-p53 proteins are present in tetraploid TS cells prior to differentiation and that their levels do not change after differentiation. Interestingly, the level of *Bax* transcription in tetraploid TS cells was consistently maintained at very low levels, indicating that p53 could not activate the *Bax* gene; thus the p53-dependent tetraploidy checkpoint will not function in the trophoblast lineage due to very low expression of the p53 effector, Bax.

There are several reports that support the hypothesis underlying the lineage-specific tetraploidy checkpoint. In general, undifferentiated TS cells proliferate in the presence of FGF4 and in medium conditioned by mouse embryonic fibroblasts by forming tightly packed colonies; however, once the FGF4 or conditioned medium is removed, the TS cells spontaneously differentiate into trophoblast giant (TG) cells that increase in size and show genome endoreduplication^37^. The DNA content of differentiated TG cells generally ranges from 8n to 64n, indicating that TG cells are resistant to polyploidisation. One of the key regulators to polyploidise TS cells is cyclin-dependent protein kinase 1 (CDK1), which is the enzyme that enables cells to enter mitosis. Inactivation of CDK1 is necessary for TS cell differentiation. Artificial inhibition of CDK1 induces the differentiation of TS cells into polyploid TG cells; however, CDK1 inhibition in ES cells induces abortive endoreduplication and apoptosis^38^, revealing that inactivation of CDK1 triggers endoreduplication only in cells that are programmed to differentiate into polyploid cells. In addition, it is worth noting that epiblast cells, but not extraembryonic cells, at the egg cylinder stage are highly sensitive to DNA damage, which stimulates p53-dependent apoptosis^39^. As described above, aberrant mitosis in tetraploid cells can cause DNA damage, which then triggers G1 arrest and cell death^29^. These reports suggest that trophoblast cells are able to tolerate p53-dependent apoptosis initiated upon tetrapolyploidisation.

The tetraploid-diploid chimera experiment generated chimeras with 100% (or very nearly) of tetraploid cells. Although the proliferation rate of tetraploid and diploid ES cells was similar (Fig. S1E,F), it is not clear why only chimeras with tetraploid cells were generated. In general, p53-deficient cells proliferate faster than wild-type cells^36^; therefore, p53-deficient tetraploid cells, which had overcome apoptosis, could have survived the competition with diploid cells during mid- and late-gestation. Although it is unclear which tissues were rescued by diploid cells during mid-gestation, diploid cells may be necessary for tetraploid development after E14.5 in mice. These tetraploid chimeras developed to term; however, the 100% or high contribution of tetraploid chimeras die soon after birth. The reason for this is unknown now; however, increase and/or unbalance of gene expression level by the duplication of genome in tetraploid embryos could cause postnatal death of tetraploid chimeras.

It is possible that p53 downregulation improves not only tetraploid development but also uniparental development; for example, the development of parthenogenetic embryos, which die due to the miss-expression of imprinted genes^40^. However, the development of parthenogenetic embryos was not rescued by p53 downregulation. p53-downregulated parthenogenetic embryos did not develop beyond E9.5, at which point wild-type parthenogenetic embryos stopped developing (0/40, 0%), indicating that the p53 downregulation-mediated improvement in development was specific to tetraploid embryos.

We also showed that tetraploid mice carrying wild-type p53 were embryonic lethal, and that only p53-downregulated embryos survived. However, tetraploidy is common in fish and amphibians, although they have a functional p53 protein. For example, *Xenopus* p53 is biochemically similar to mammalian p53 and is induced upon DNA damage in somatic cells^41^. Both *Xenopus* and mammalian p53 function as a tumour suppressor, preventing aberrant genomic changes such as tetraploidy, which can occur in cancer cells. By contrast, the role of p53 during development is different in *Xenopus* and mammals. p53-deficient mice are developmentally normal and develop to term^42^, whereas inhibiting p53 function in *Xenopus* results in an early block on differentiation^43^. Therefore, p53 in vertebrates appears to be evolving still in functional terms, and the protein shows some functional differences between species during early development. Although tetraploidy has generated new genes as a result of whole genome duplication events, which have helped to drive evolution, one of these genes, *p53*, suppresses tetraploidy, suggesting that tetraploid evolution itself is an evolutionary dead-end. The results of the present study provide new insights into the relationship between tetraploid evolution and newly-evolved genes.

In conclusion, this study demonstrated that tetraploidy in differentiating epiblast and ES cells triggered p53-dependent apoptosis, indicating the activation of a tetraploidy checkpoint during early development. We also showed that downregulation of p53 rescued tetraploid development in mice. However, p53 is not the only factor that regulates tetraploidy survival because tetraploid mice died immediately after birth. Therefore, it will be necessary to identify other factors that affect the fate of tetraploid mammals.

## Methods

### Mice

C57BL/6-Tg (ACTbEGFP) 1 Osb/J (B6-EGFP) mice were the kind gift of Dr. M. Okabe. C57BL/6(B6)-*p53*^+/−^ mice (CDB 0001K) that do not express p53 protein since a neomycin resistance gene is placed in the second exon of the *p53* gene, and PWK (RBRC00213) mice were provided by the RIKEN BioResource Center (BRC) through the National Bio-Resource Project, MEXT, Japan^44^. CD1(ICR) and 129Xl/SVJ (129) mice were purchased from Charles River Japan and Japan SLC Inc., respectively. All animal experiments were approved by the guidelines of the Animal Care and Experimentation Committee of Gunma University, Showa Campus, Japan (No. 11-007) and in accordance with the approved guidelines of Gunma University.

### Preparation of Tetraploid Embryos by Electrofusion

Females were superovulated by injecting 5 units of pregnant mare's serum (PMSG; ASKA Pharmaceutical, Tokyo, Japan) followed 48 h later with 5 units of human chorionic gonadotropin (hCG; ASKA Pharmaceutical). After administration of hCG, females were mated with males. Fertilised zygotes from B6-EGFP × PWK and B6/129 F1-*p53*^+/−^ × B6/129 F1-*p53^+/−^* mice were isolated from the oviduct 40–42 h later. After washing in M2 medium, zygotes were transferred to drops of M16 medium at 37°C. Forty-two hours post-hCG treatment, the blastomeres of two-cell embryos were electrofused to produce tetraploid embryos. Electrofusion was carried out in a 400 μl drop of 0.3 M Mannitol medium supplemented with 0.5 mM CaCl_2_ and 0.1 mM MgSO_4_ by a single electrical pulse of 7 V with a duration of 100 μs. After electrofusion, embryos were returned to M16 medium at 37°C and cultured to blastocyst stage.

### Generation of ES and TS cells

To generate ES cells, tetraploid and diploid blastocysts were transferred into gelatinised tissue culture wells and cultured in ES medium (DMEM containing 17.5% Knockout SR; Gibco, Gland Island, NY) according to standard procedures^45^. ICM outgrowths were harvested in 0.25% Trypsin/1 mM EDTA and then passaged prior to freezing or use. To generate TS cells, blastocysts were cultured on a feeder layer of primary mouse embryonic fibroblast cells in TS cell medium containing FGF4 and heparin following standard procedures^36^. Trophoblast outgrowths were harvested in 0.1% Trypsin/1 mM EDTA and then passaged prior to freezing or use.

### Production of tetraploid mice and tetraploid-diploid chimeras

Tetraploid-diploid chimeric embryos were produced by injecting five to ten WBB6F1-*W/W^v^* diploid ES cells (male line) or BPF1-GFP diploid ES cells (female line) into the blastocoel cavity of p53-deficient tetraploid blastocysts. Tetraploid and chimeric blastocysts were transferred to the uterine horns of pseudopregnant recipient females at 2.5 days post coitus. Embryos after the blastocyst stage were placed into a foster mother and then harvested at the appropriate embryonic stage.

### Differentiation of ES and TS cells

To induce embryoid body (EB) formation from ES cells, ES cells were detached and dissociated into single cells with 0.25% Trypsin/1 mM EDTA and then plated on a 10 cm bacterial culture dish in 10 ml of DMEM supplemented with 10% fetal bovine serum (Hyclone, Logan, UT), nonessential amino acids (0.1 mM) and 2-mercaptoethanol (0.1 mM). To differentiate TS cells into trophoblast giant cells, TS cells were cultured in TS cell medium lacking FGF4 and heparin for 5 days. The medium was changed every 2 days.

### FACS Analysis

Detection of active caspases was conducted using an Apoptosis Detection Kit Caspase Assay (Immunochemistry Technologies, Bloomington, MN). Briefly, trypsinised cells were labelled with SR-VAD-FMK for 1 h at 37°C, washed in 1× apoptosis wash buffer and placed on ice in the dark. SR-VAD-FMK is labeled with sulforhodamine B, a red fluorescent dye with optimal excitation at 565 nm and emission at 590–600 nm. Caspase activity was detected using a FACSCalibur HG flow cytometer (Becton Dickinson, Franklin Lakes, NJ) and an emission filter associated with the FL-2 channel.

### Detection of Apoptosis

A 200 ng DNA solution was loaded on a 1.0% agarose gel for electrophoresis and the resulting fragments were observed with an UV transilluminator.

### Quantitative real-time RT-PCR

Total RNA purified from ES and TS cells was reverse transcribed using Superscript II (TaKaRa, Otsu, Japan) and an oligo(dT)12–18 primer (TaKaRa) in a total volume of 20 μl. Quantitative real-time RT-PCR was performed using SYBR Premix Ex Taq (Perfect Real Time, TaKaRa). The PCR mixture consisted of 2 × SYBR Premix Ex Taq, 10 μM forward and reverse primers, and template cDNA in a total volume of 12.5 μl. The cocktail was activated by heating at 95°C for 10 sec. The subsequent PCR reaction was carried out at 95°C for 5 sec and 60°C for 30 sec for 40 cycles in a LightCycler480 (Roche). PCR amplification was performed using the following primer sets:

*p53*: 5′-GTCACGCTTCTCCGAAGACT-3′

and 5′-GTCCATGCAGTGAGGTGATG-3′

*Bax*: 5′-GCTGGACACTGGACTTCCTC-3′

and 5′-GAGGACTCCAGCCACAAAGA-3′

*Bcl-2*: 5′-AGTACCTGAACCGGCATCTG-3′

and 5′-GCTGAGCAGGGTCTTCAGAG-3′

*Oct4*: 5′-CCAATCAGCTTGGGCTAGAG-3′

and 5′-CTGGGAAAGGTGTCCCTGTA-3′

*Nanog*: 5′- ATGCCTGCAGTTTTTCATCC-3′

and 5′- GAGGCAGGTCTTCAGAGGAA-3′

*Sox2*: 5′- GAGTGGAAACTTTTGTCCGAGA-3′

and 5′- GAAGCGTGTACTTATCCTTCTTCAT-3′

*Stella*: 5′- AGCCGTACCTGTGGAGAACAA-3′

and 5′-TCTTTCAGCACCGACAACAAA-3′

*Fgf5*: 5′-CTCAGGGGATTGTAGGAATACGAGGA-3′

and 5′-GGATCGCGGACGCATAGGTATTATAG-3′

*Gapdh*: 5′-AATGCATCCTGCACCACCAA-3′

and 5′-GTGGCAGTGATGGCATGGAC-3′

The *Gapdh* gene was used to standardise the data.

### Immunoblot analysis

Whole cell extracts from ES and TS cells were subjected to 10% SDS-PAGE gel electrophoresis (1 × 10^5^ cells/lane for p53 and p-p53, and 5 × 10^3^ cells/lane for α-tubulin) and the proteins transferred to a PVDF membrane. The following antibodies were used for immunoblotting: anti-p53 (1:2,000, CM5; Leica Microsystems, Wetzlar, Germany), anti-phopho-p53 (Ser15) (1:2,000, #9284; Cell Signaling Technology, Danvers, MA) and anti-α-tubulin (1:4,000, PM054; MBL, Nagoya, Japan). Signals were detected by chemiluminescence (ECL Prime; GE healthcare, Buckinghamshire, UK) and a CCD camera (LAS-4000, Fujifilm, Tokyo, Japan). The intensity of the protein bands was quantified using Image J software (NIH).

### Immunocytochemistry and TUNEL assay

Embryos were dissected from the maternal decidua, fixed with 4% paraformaldehyde/PBS at 4°C overnight and then immunostained with an anti-p53 antibody (1:500, CM5) followed by an anti-rabbit IgG fluorescein-conjugated antibody (1:500, ICN 55354; MP Biomedicals, Solon, OH). TUNEL was performed using the *In Situ* Cell Death Detection Kit (Roche, Mannheim, Germany).

### Chromosome analysis

Chromosome analysis by Q-banding was performed by the Central Institute for Experimental Animals (CIEA; Kawasaki, Japan) using epithelial cells derived from back skin.

### Chimerizm analysis

For tetraploid chimeras generated by the introduction of *W/W^v^* diploid ES cells, quantitative PCR analysis was performed to detect *W^v^* mutant and wild-type cells as previously reported^46^. If the *W^v^* type was not amplified but the wild type was, the contribution of diploid cells was considered to be <0.1%. For tetraploid chimeras that were generated by the introduction of GFP-positive diploid ES cells, tissues were dissociated in trypsin/EDTA and the percentage of GFP-negative cells (tetraploid cells) was calculated.

### Statistical analysis

Data are shown as means and standard deviations. The Student's t-test (two-tailed test) was used for cell size, cell growth and gene expression analyses, and a p-value of <0.05 was considered significant.

## Author Contributions

T.H. designed and performed experiments, and wrote the manuscript. M.Y. collected embryos and discussed the results. S.M. and M.K. performed DNA isolation and genotyping of mice. Y.N. established parts of ES cell lines and discussed the results. I.H. supervised the project and wrote the manuscript.

## Supplementary Material

Supplementary Information Supplementary Information

## Figures and Tables

**Table 1:** Postimplantation development of tetraploid embryos transferred to pseudopregnant recipients analysed at various stages of gestation

		No.	No.	No.	No. embryos	No. embryos	No. embryos *p53*		
		embryos	implants	resorption	recovered	alive**	*+/+*	*+/−*	*−/−*
Days Caesarian of section*		transferred	(% transferred)	(% implants)	(% implants)	(% implants)	(%examined)		
Tetraploid	7.5	50	ND	ND	27		7 (25.9)	14 (51.8)	6 (22.2)
	8.5	27	18 (66.7)	4 (22.2)	14 (77.8)		4 (28.6)	5 (35.7)	5 (35.7)
	10.5	40	32 (80.0)	25 (78.1)	7 (21.8)	7 (21.8)	0 (0)	4 (57.1)	3 (42.8)
	12.5	50	45 (90.0)	30 (66.6)	15 (33.3)	13 (28.8)	0 (0)	7 (53.8)	6 (46.1)
	14.5	55	37 (67.2)	29 (78.3)	8 (21.6)	4 (10.8)	0 (0)	5 (62.5)	3 (37.5)
	15.5	20	16 (80.0)	13 (81.2)	3 (18.7)	0 (0)	0 (0)	0 (0)	1 (100.0)
	19.5	22	18 (81.8)	12 (66.6)	6 (33.3)	0 (0)	0 (0)	0 (0)	0 (0)
Diploid	12.5	15	13 (86.6)	1 (7.6)	12 (92.3)	12 (92.3)	4 (33.3)	5 (41.6)	3 (25.0)
	19.5	Natural mating					19 (39.5)	21 (43.7)	8 (16.6)

*Tetraploid blastocysts were transferred to the 2.5 dpc pseudopregnant recipients.

**Heart beating at time isolation.

ND, not determined.

**Table 2:** Generation of tetraploid-diploid chimeras

Diploid ES cell line	Chimera ID	Body weight	Sex	Tetraploid contribution
WBB6F1-W/Wv (male)	110416CS2	1.24 g	male	100% (10 tissues, Fig. 6A)
	111109CS1	1.26 g	hermaphroditism	22 ~ 68% (10 tissues)
BPF1-GFP (female)	110522CS2	1.48 g	female	about 10% (coat color)
	110522CS4	2.31 g	male	4 ~ 74% (10 tissues, Fig. 6E)
	110711CS1	1.12 g	male	14% (tail tips)
	110802CS1	not determined	male	89% (tail tips)
