# Supplementary material for: p53 Suppresses Tetraploid Development in Mice
Source: Sci Rep. 2015 Mar 10;5:8907. doi: 10.1038/srep08907 (PMC4354145; doi:10.1038/srep08907)
Supplement: Supplementary Information [file srep08907-s1.pdf]

SUPPLEMENTARY INFORMATION

Title: p53 Suppresses Tetraploid Development in Mice

Authors: Takuro Horii, Masamichi Yamamoto, Sumiyo Morita, Mika Kimura, Yasumitsu Nagao, Izuho Hatada*

Supplementary Methods

Ploidy measurement

For chromosome analysis, tetraploid and diploid cells were exposed to colcemid at a concentration of 0.03 μg/ml for 3 h. The cells were trypsinised, swelled with 0.075 M KCl, fixed with ice-cold acetic acid/methanol (1:3, v/v), and then dropped onto glass slides. The cell-chromosome mixture was stained with DAPI (4', 6-diamino-2-phenylindole) solution to visualise the chromosomes for fluorescence microscopy. For cell cycle analysis, cells were treated using the Cell Cycle Phase Determination Kit (Cayman chemical, Ann Arbor, MI), and then were analysed using a BD FACSCalibur HG flow cytometer.

DNA methylation analysis

DNA was isolated from each ES cell line and from EBs. Bisulfite treatment was carried out using an Epitect Bisulfite kit (Qiagen, Hilden, Germany), according to the manufacturer’s instructions. PCR amplification of *Oct4* proximal enhancer (PE) and promoter and *Nanog* tissue-dependent and differentially methylated region (T-DMR) was performed for each set of isolated cells using the following primer sets:

Oct4 PE and promoter: 5’-CCACCCTCTAACCTTAACCTCTAAC-3’
and 5’-TGAGGAGTGGTTTTAGAAATAATT-3’

Nanog T-DMR: 5’-TTTGTAGGTGGGATTAATTGTGAAT-3’
and 5’-AAAAAAACAAAACACCAACCAAAT-3’

The amplification consisted of a total of 38 cycles at 94°C for 10 sec, 55°C for 30 sec, and 72°C for 60 sec in a GeneAmp PCR system 9700 (Applied Biosystems, Foster City, CA).

PCR products were subcloned into the TA cloning vector (pCR 2.1; Invitrogen). Positive clones in each sample were sequenced using the Big Dye terminator method (ABI PRISM 3100; Applied Biosystems). For COBRA, *Oct4* PE and promoter PCR products were digested using HpyCH4 IV. Next, DNA fragments were separated on a 2% agarose gel, and DNA band intensities were measured using Image J software (NIH), and methylation percentages were calculated.

Supplemental Figure Legends

Figure S1. Ploidy, cell size and growth of tetraploid and diploid ES cells. (A, B) Chromosome analysis and (C, D) cell cycle analysis clarified that both tetraploid (A, C) and diploid (B, D) ES cells maintained ploidy during *in vitro* culture (passage 5). (E) The diameter of tetraploid ES cells is significantly larger than that of diploid ES cells (T-test, $p < 0.05$) due to greater DNA content. (F) Doubling times of tetraploid ES cells were almost equivalent to diploid ES cells, thus tetraploid embryo developmental defects are not due to differences in proliferation speed.

Figure S2. Comparison of pluripotency markers between tetraploid and diploid ES cells. Quantitative real-time RT-PCR analyses of pluripotency marker expression in tetraploid and diploid ES cells under undifferentiated conditions. *Genes showing significant differences between tetraploid and diploid ES cells (T-test, $p < 0.05$). Tetraploid ES cell lines: 4n1–4n4; diploid ES cell lines, 2n1–2n4.

Figure S3. Immunoblots analysis of p53, p-p53(Ser18), and tubulin expression in ES and TS cells. For p53 and p-p53, 1×10^5 cells were loaded into each lane; for tubulin, 5×10^3 cells were loaded into each lane.

Figure S4. Tetraploid ES cells that survived differentiation treatment are mostly undifferentiated. (A) Relative mRNA expression levels of pluripotency markers (*Oct4* and *Nanog*) in tetraploid ES cells were not sufficiently downregulated after differentiation treatment. (B) Results of combined bisulfite restriction analysis (COBRA) and (C) bisulfite sequencing for promoter and/or enhancer regions at d12 EB showing hypomethylation of *Oct4* and *Nanog* in surviving tetraploid cells. Tetraploid ES cell lines, 4n1–4n2; diploid ES cell lines, 2n1–2n2. Open circles, unmethylated CpG; closed circles, methylated CpG.

Figure S5. TUNEL staining for p53-deficient tetraploid embryos at E5.5-7.5. TUNEL positive cells were reduced in these $p53^{-/-}$ and $p53^{+/-}$ embryos. Scale bars: 0.1 mm.

Figure S6. p53-deficient tetraploid embryos maintain tetraploidy. (A, B) Representative chromosomes and (C, D) histograms showing chromosome numbers for tetraploid male (A, C) and female (B, D) embryos at E14.5.

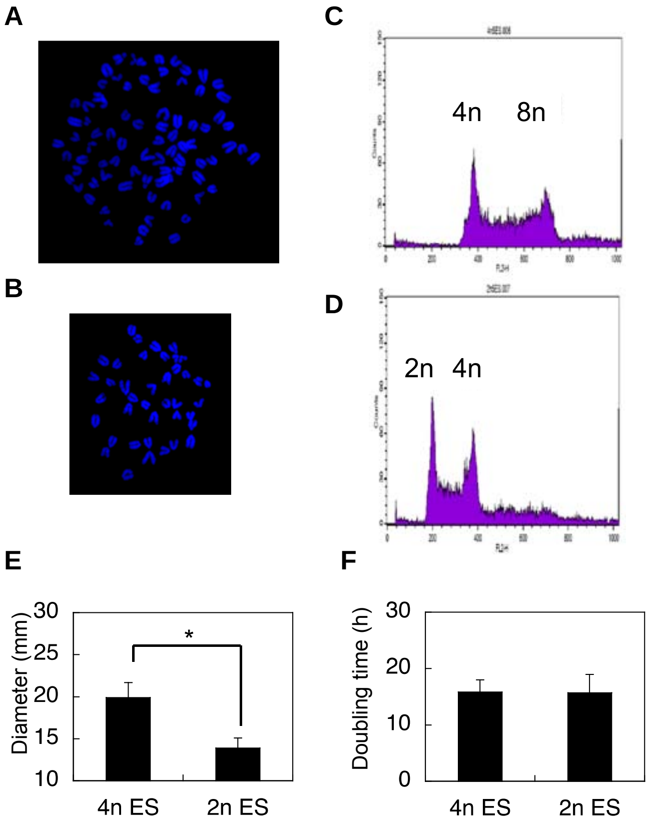

Supplementary Figure S1

Horii et al., 2014

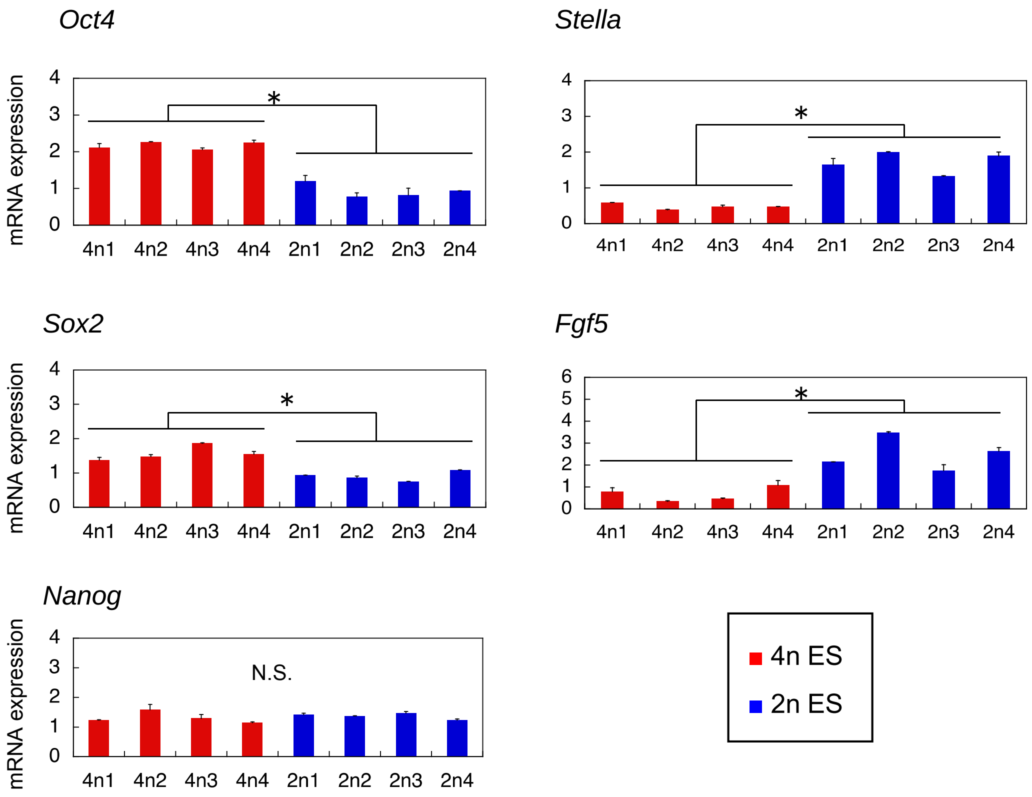

Supplementary Figure S2

Horii et al., 2014

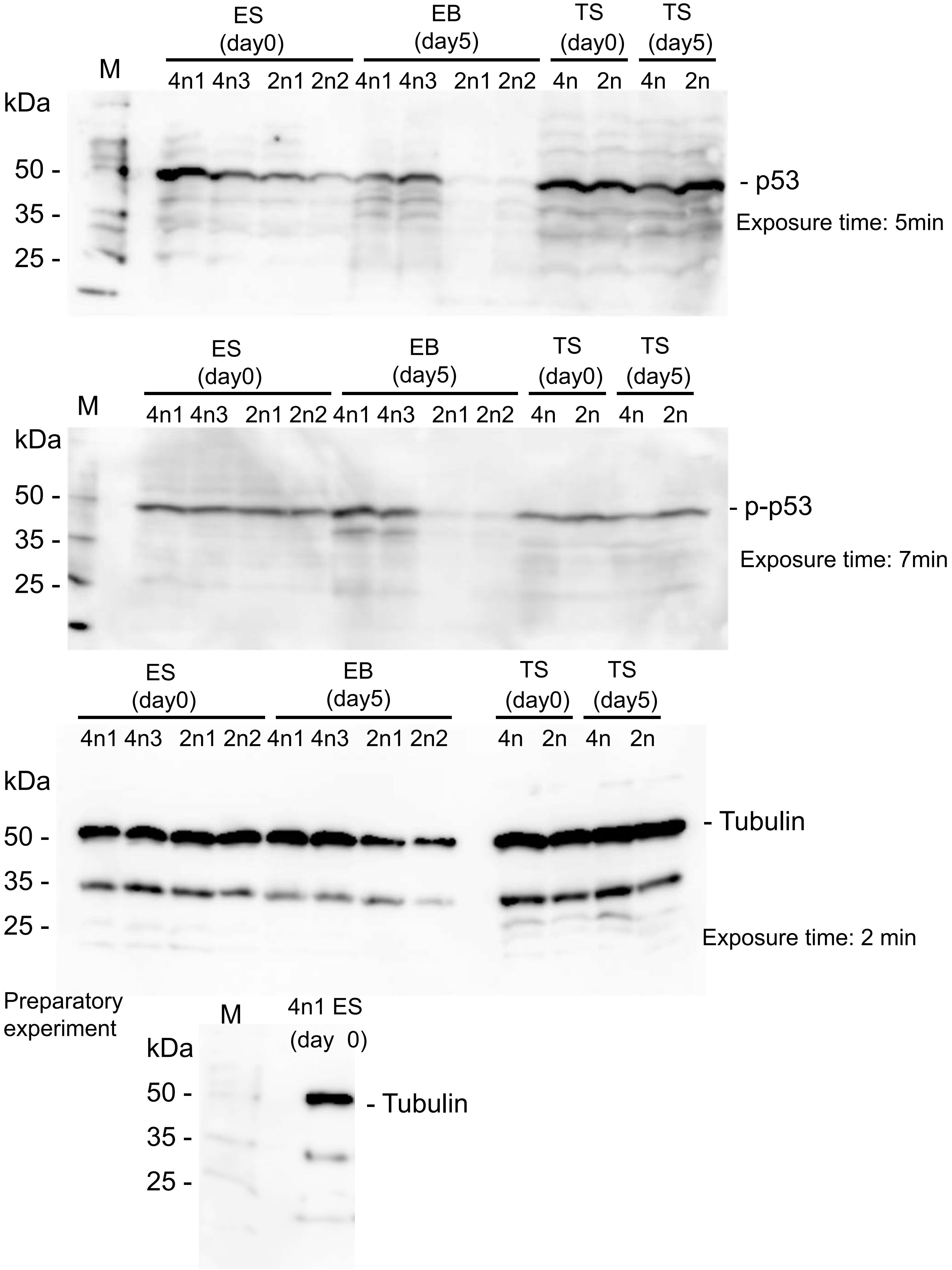

Supplementary Figure S3

Horii et al., 2014

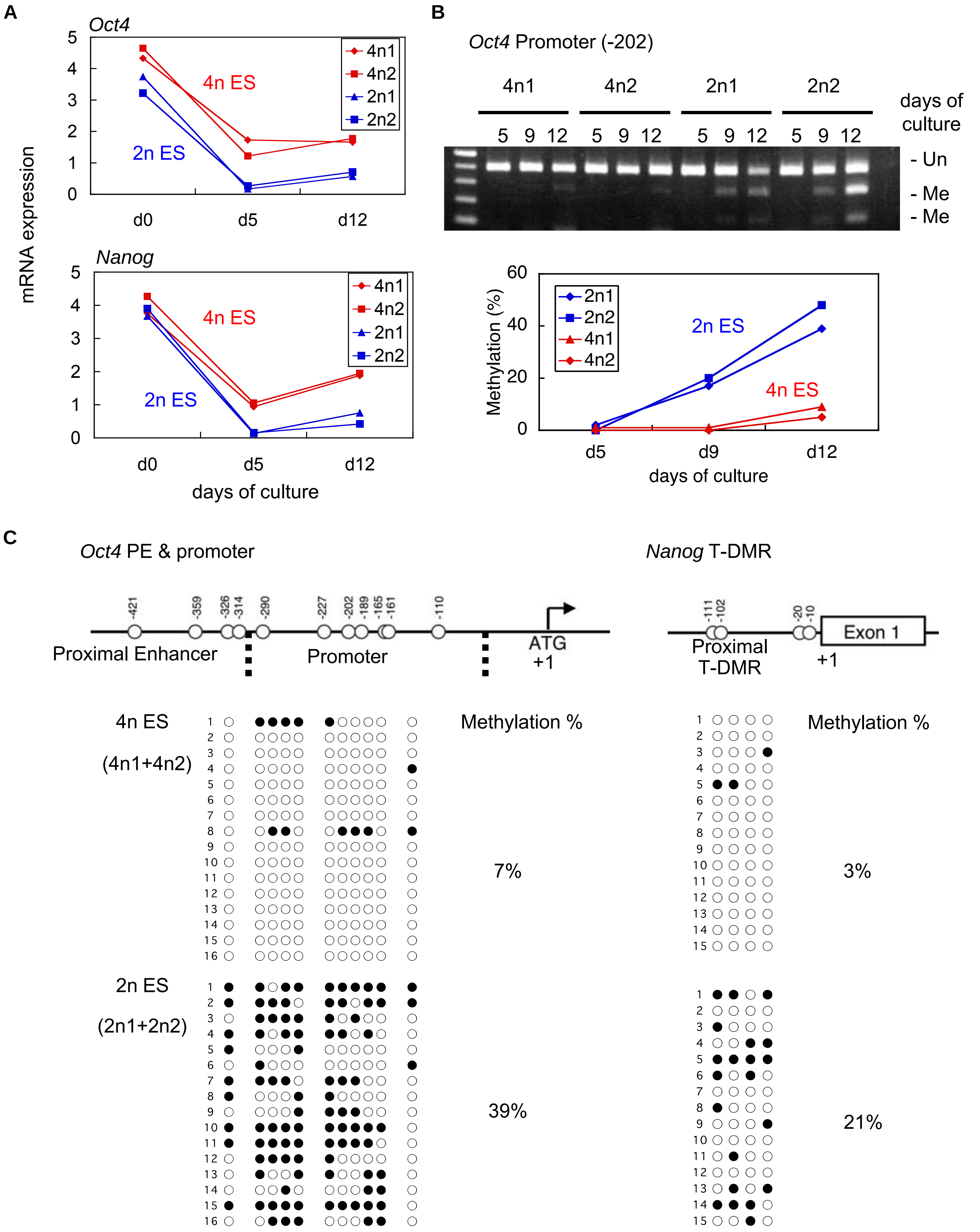

Supplementary Figure S4

Horii et al., 2014

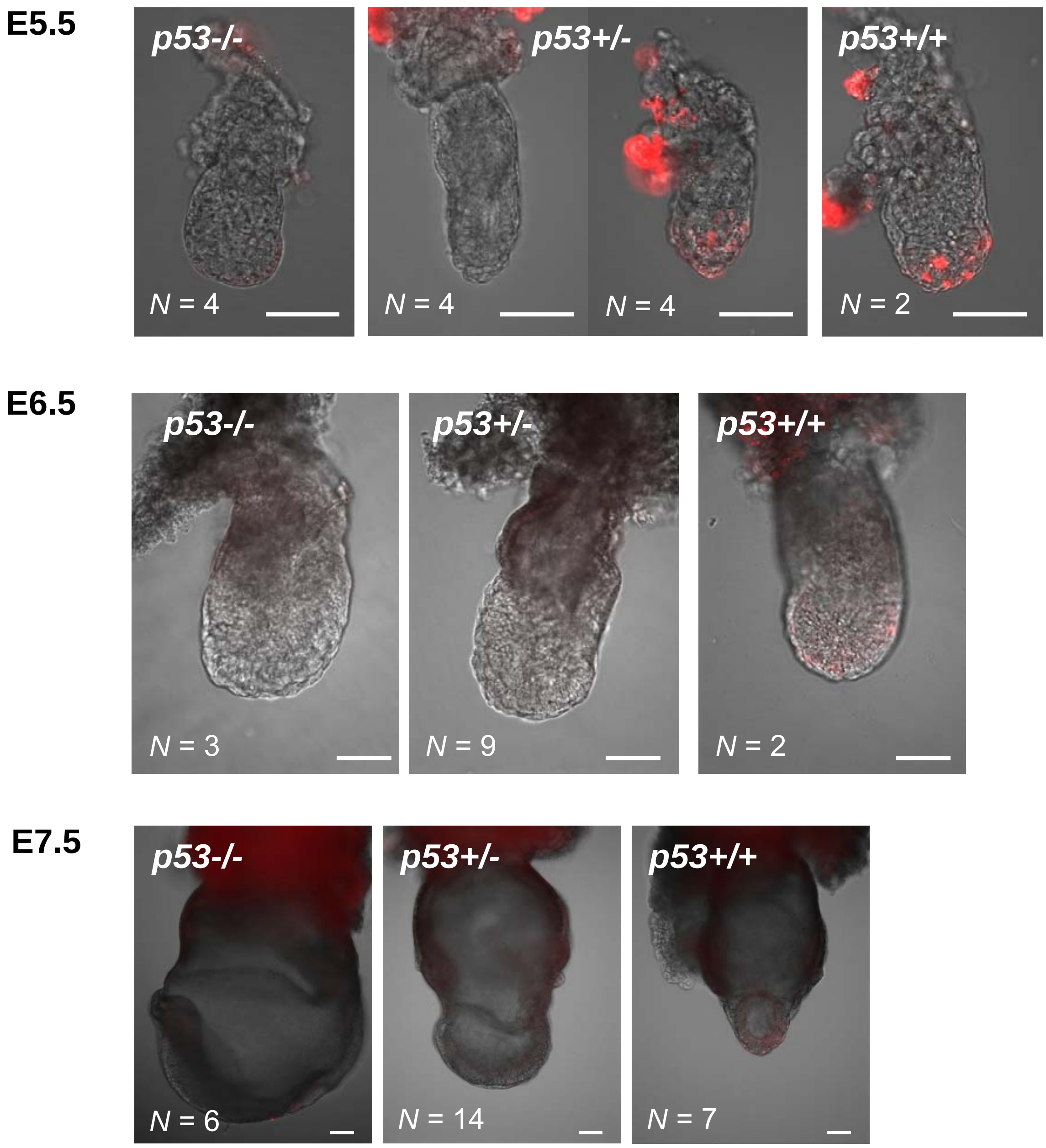

Supplementary Figure S5

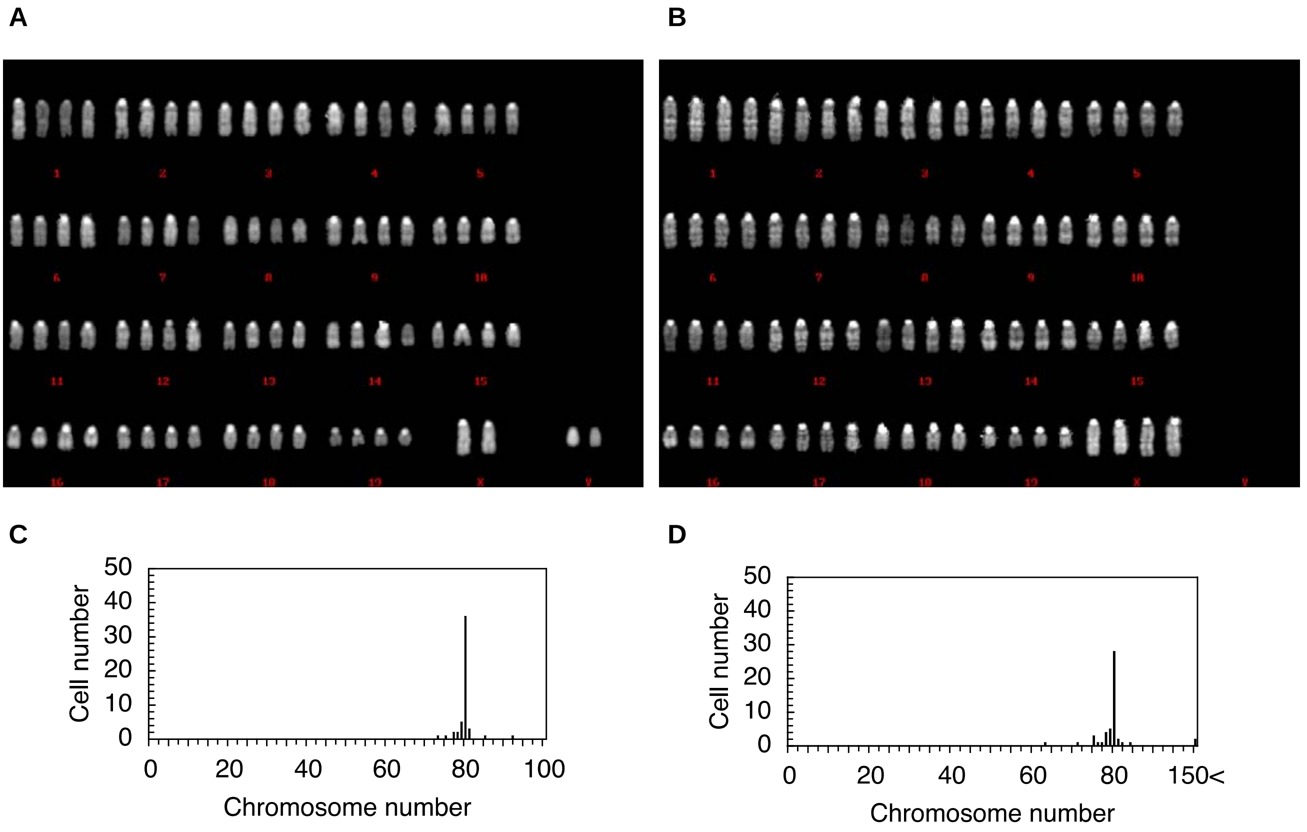

Supplementary Figure S6

Horii et al., 2014
